# 1-[(3*RS*,4*RS*)-1-Benzyl-4-methyl­piperi­din-3-yl]-1,6-dihydro­imidazo[4,5-*d*]pyrrolo­[2,3-*b*]pyridine hemihydrate

**DOI:** 10.1107/S1600536812039980

**Published:** 2012-09-29

**Authors:** Ellen Pfaffenrot, Dieter Schollmeyer, Stefan Laufer

**Affiliations:** a Eberhard-Karls-University Tübingen, Auf der Morgenstelle 8, 72076 Tübingen, Germany; b Institute of Organic Chemistry, University Mainz, Duesbergweg 10-14, 55099 Mainz, Germany

## Abstract

The benzyl residue in the title compound, C_21_H_23_N_5_·0.5H_2_O, is oriented at a dihedral angle of 83.8 (3)° towards the 1,6-dihydro­imidazo[4,5-*d*]pyrrolo­[2,3-*b*]pyridine system. The piperidine ring adopts a chair conformation with the *cis* substituents displaying a torsion angle of −45.91 (16)°. In the crystal, mol­ecules are accumulated as racemic dimers by two inter­molecular hydrogen bonds between the pyrrolo­pyridine systems. Another hydrogen bond is formed between the imidazole ring and the cocrystallized water mol­ecule, which is located on a twofold rotation axis.

## Related literature

For biological details on Janus protein tyrosine kinases, see: Kulagowski *et al.* (2012 ▶). For synthetic details, see: Bajwa *et al.* (2006 ▶).
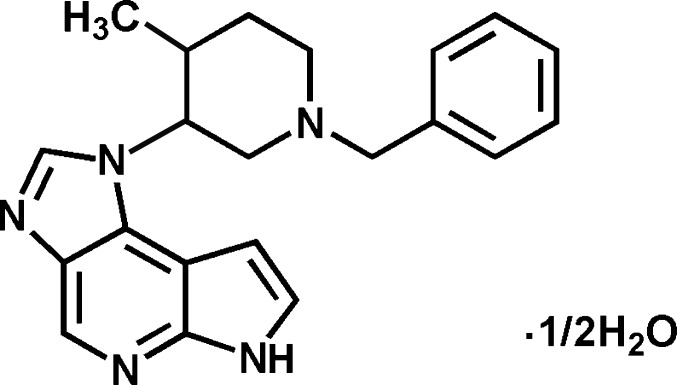


## Experimental

### 

#### Crystal data

C_21_H_23_N_5_·0.5H_2_O
*M* *_r_* = 354.45
Monoclinic, 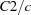

*a* = 17.3606 (19) Å
*b* = 10.0422 (10) Å
*c* = 22.995 (2) Å
β = 100.965 (3)°
*V* = 3935.8 (7) Å^3^
*Z* = 8
Mo *K*α radiation
μ = 0.08 mm^−1^
*T* = 173 K
0.50 × 0.27 × 0.20 mm

#### Data collection

Bruker APEXII diffractometer
22115 measured reflections
4571 independent reflections
3504 reflections with *I* > 2σ(*I*)
*R*_int_ = 0.040
Standard reflections: ?

#### Refinement

*R*[*F* ^2^ > 2σ(*F* ^2^)] = 0.045
*wR*(*F* ^2^) = 0.109
*S* = 1.03
4571 reflections
240 parameters
H-atom parameters constrained
Δρ_max_ = 0.37 e Å^−3^
Δρ_min_ = −0.30 e Å^−3^

### 

Data collection: *APEX2* (Bruker, 2006 ▶); cell refinement: *APEX2*; data reduction: *APEX2*; program(s) used to solve structure: *SIR97* (Altomare *et al.*, 1999 ▶); program(s) used to refine structure: *SHELXL97* (Sheldrick, 2008 ▶); molecular graphics: *PLATON* (Spek, 2009 ▶); software used to prepare material for publication: *PLATON*.

## Supplementary Material

Crystal structure: contains datablock(s) I, global. DOI: 10.1107/S1600536812039980/bt6840sup1.cif

Structure factors: contains datablock(s) I. DOI: 10.1107/S1600536812039980/bt6840Isup2.hkl

Supplementary material file. DOI: 10.1107/S1600536812039980/bt6840Isup3.cml

Additional supplementary materials:  crystallographic information; 3D view; checkCIF report

## Figures and Tables

**Table 1:** Hydrogen-bond geometry (Å, °)

*D*—H⋯*A*	*D*—H	H⋯*A*	*D*⋯*A*	*D*—H⋯*A*
N8—H8⋯N6^i^	0.98	2.02	2.9757 (18)	165
O1*W*—H1*W*⋯N3	0.93	2.13	3.0148 (16)	161

Symmetry code: (i) 
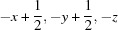
.

**Fig. 1.**
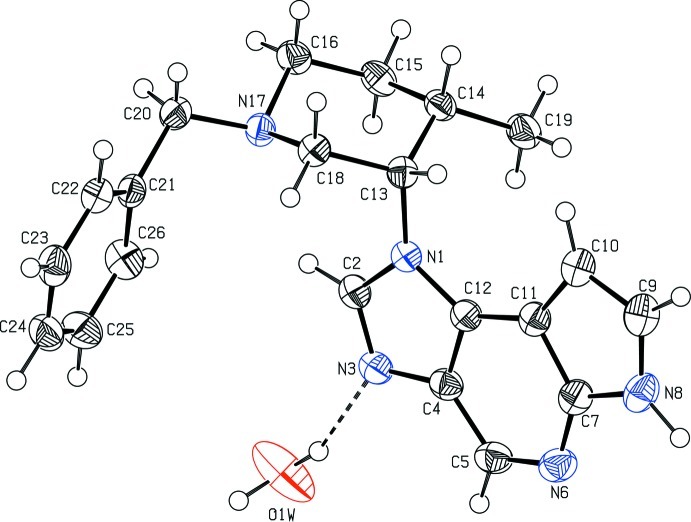
Crystal structure of the title compound with labelling and displacement ellipsoids drawn at the 50% probability level. Hydrogen bond is represented as dashed lines.

## supplementary crystallographic information

### Comment

Imidazopyrrolopyridine derivates were identified as novel tricyclic JAK inhibitors. The Janus protein tyrosine kinases (JAK1, JAK2, JAK3 and TYK2) regulate the signal transduction of numerous cytokines, presenting a key role in immune and inflamatory processes (Kulagowski *et al.*, 2012).

The planar imidazopyrrolopyridine system is oriented at a dihedral angle of 83.8 (3)° and shows a distance of 6.89 (8) Å with respect to the benzyl group (Fig. 1). The equatorial methyl and the axial tricyclic substituent of the piperidine ring show a torsion angle of 45.9 (1)°. The crystal structure is characterized by two types of intermolecular hydrogen bonds. The water molecule connects two by 2-fold axis related pyrrolopyridine systems (O1W–H1W···N3 2.13 Å) while the N8—H8···N6 hydrogen bond build centrosymmetric dimers (Tabl. 1).

### Experimental

The title compound was prepared by deprotection of a *N*-tosylated precursor (Bajwa *et al.*, 2006). 1-(1-benzyl-4-methylpiperidin-3-yl)-6-tosyl-1,6-dihydro-imidazo[4,5-*d*]pyrrolo[2,3-*b*]pyridine (0.164 g, 0.329 mmol) and caesium carbonate (0.986 g, 0.321 mmol) were dissolved in a mixture of dry THF (2 ml) and dry MeOH (1 ml) under argon atmosphere. The mixture was stirred for 3.5 h at room temperature and the reaction monitored by HPLC. When the reaction was complete, saturated NaHCO_3_ solution was added and the mixture was extracted with ethylacetate. The organic phase was dried with anhydrous Na_2_SO_4_ and concentrated *in vacuo*. The precipitated product was obtained as crystalline solid by filtration (0.091 g, 80%). Crystals of the title compound were obtained by slow evaporation of methanol at room temperature.

### Refinement

Hydrogen atoms attached to carbon and nitrogen atoms were placed at calculated positions with C—H = 0.95 Å (aromatic) or 0.99–1.00 Å (*sp*^3^ C-atom). The position of the water H atom was taken from a difference map. All H atoms were refined using a riding model with isotropic displacement parameters set at 1.2–1.5 times of the *U*_eq_ of the parent atom.

### Crystal data

**Table:**

C_21_H_23_N_5_·0.5H_2_O	*F*(000) = 1512
*M**_r_* = 354.45	*D*_x_ = 1.196 Mg m^−^^3^
Monoclinic, *C*2/*c*	Mo *K*α radiation, λ = 0.71073 Å
Hall symbol: -C 2yc	Cell parameters from 4892 reflections
*a* = 17.3606 (19) Å	θ = 2.4–26.7°
*b* = 10.0422 (10) Å	µ = 0.08 mm^−^^1^
*c* = 22.995 (2) Å	*T* = 173 K
β = 100.965 (3)°	Block, colourless
*V* = 3935.8 (7) Å^3^	0.50 × 0.27 × 0.20 mm
*Z* = 8	

### Data collection

**Table:**

Bruker APEXII diffractometer	3504 reflections with *I* > 2σ(*I*)
Radiation source: sealed Tube	*R*_int_ = 0.040
Graphite monochromator	θ_max_ = 27.7°, θ_min_ = 1.8°
CCD scan	*h* = −21→22
22115 measured reflections	*k* = −13→12
4571 independent reflections	*l* = −30→30

### Refinement

**Table:**

Refinement on *F*^2^	Primary atom site location: structure-invariant direct methods
Least-squares matrix: full	Secondary atom site location: difference Fourier map
*R*[*F*^2^ > 2σ(*F*^2^)] = 0.045	Hydrogen site location: inferred from neighbouring sites
*wR*(*F*^2^) = 0.109	H-atom parameters constrained
*S* = 1.03	*w* = 1/[σ^2^(*F*_o_^2^) + (0.0401*P*)^2^ + 2.9543*P*] where *P* = (*F*_o_^2^ + 2*F*_c_^2^)/3
4571 reflections	(Δ/σ)_max_ < 0.001
240 parameters	Δρ_max_ = 0.37 e Å^−^^3^
0 restraints	Δρ_min_ = −0.30 e Å^−^^3^

### Special details

**Table:**

Geometry. All e.s.d.'s (except the e.s.d. in the dihedral angle between two l.s. planes) are estimated using the full covariance matrix. The cell e.s.d.'s are taken into account individually in the estimation of e.s.d.'s in distances, angles and torsion angles; correlations between e.s.d.'s in cell parameters are only used when they are defined by crystal symmetry. An approximate (isotropic) treatment of cell e.s.d.'s is used for estimating e.s.d.'s involving l.s. planes.
Refinement. Refinement of *F*^2^ against ALL reflections. The weighted *R*-factor *wR* and goodness of fit *S* are based on *F*^2^, conventional *R*-factors *R* are based on *F*, with *F* set to zero for negative *F*^2^. The threshold expression of *F*^2^ > σ(*F*^2^) is used only for calculating *R*-factors(gt) *etc*. and is not relevant to the choice of reflections for refinement. *R*-factors based on *F*^2^ are statistically about twice as large as those based on *F*, and *R*- factors based on ALL data will be even larger.

### Fractional atomic coordinates and isotropic or equivalent isotropic displacement parameters (Å^2^)

**Table:**

	*x*	*y*	*z*	*U*_iso_*/*U*_eq_	
N1	0.23581 (6)	0.09700 (11)	0.24008 (5)	0.0258 (3)	
C2	0.30258 (8)	0.02507 (14)	0.26224 (7)	0.0293 (3)	
H2	0.3148	−0.0059	0.3020	0.035*	
N3	0.34774 (7)	0.00328 (13)	0.22344 (5)	0.0310 (3)	
C4	0.30842 (8)	0.06430 (14)	0.17183 (6)	0.0288 (3)	
C5	0.33093 (9)	0.07650 (15)	0.11641 (7)	0.0338 (3)	
H5	0.3783	0.0356	0.1108	0.041*	
N6	0.28808 (8)	0.14347 (13)	0.07164 (6)	0.0362 (3)	
C7	0.22083 (9)	0.19640 (14)	0.08238 (7)	0.0319 (3)	
N8	0.16809 (8)	0.26560 (13)	0.04086 (6)	0.0373 (3)	
H8	0.1745	0.2858	0.0004	0.056*	
C9	0.10645 (10)	0.30373 (15)	0.06626 (7)	0.0375 (4)	
H9	0.0625	0.3533	0.0466	0.045*	
C10	0.11658 (9)	0.26095 (15)	0.12371 (7)	0.0328 (3)	
H10	0.0820	0.2746	0.1506	0.039*	
C11	0.18991 (8)	0.19139 (14)	0.13518 (6)	0.0285 (3)	
C12	0.23855 (8)	0.12243 (13)	0.18146 (6)	0.0261 (3)	
C13	0.17177 (8)	0.13850 (14)	0.27023 (6)	0.0257 (3)	
H13	0.1425	0.2113	0.2458	0.031*	
C14	0.11248 (8)	0.02574 (14)	0.27341 (6)	0.0284 (3)	
H14	0.0641	0.0683	0.2826	0.034*	
C15	0.14310 (8)	−0.06998 (14)	0.32409 (7)	0.0323 (3)	
H15A	0.1007	−0.1323	0.3291	0.039*	
H15B	0.1867	−0.1230	0.3139	0.039*	
C16	0.17175 (8)	0.00276 (15)	0.38202 (7)	0.0320 (3)	
H16A	0.1278	0.0524	0.3936	0.038*	
H16B	0.1915	−0.0623	0.4137	0.038*	
N17	0.23465 (7)	0.09550 (12)	0.37510 (5)	0.0275 (3)	
C18	0.20358 (8)	0.19706 (14)	0.33118 (6)	0.0273 (3)	
H18A	0.2457	0.2614	0.3279	0.033*	
H18B	0.1610	0.2463	0.3450	0.033*	
C19	0.08819 (9)	−0.04706 (17)	0.21443 (7)	0.0379 (4)	
H19A	0.0685	0.0175	0.1832	0.057*	
H19B	0.0468	−0.1116	0.2176	0.057*	
H19C	0.1337	−0.0938	0.2047	0.057*	
C20	0.26827 (9)	0.15779 (16)	0.43189 (6)	0.0331 (3)	
H20A	0.2786	0.0881	0.4629	0.040*	
H20B	0.2296	0.2207	0.4429	0.040*	
C21	0.34369 (8)	0.23169 (14)	0.43017 (6)	0.0278 (3)	
C22	0.35253 (9)	0.36471 (15)	0.44601 (6)	0.0312 (3)	
H22	0.3098	0.4116	0.4567	0.037*	
C23	0.42287 (9)	0.43037 (16)	0.44643 (7)	0.0373 (4)	
H23	0.4283	0.5213	0.4578	0.045*	
C24	0.48489 (9)	0.36375 (18)	0.43038 (7)	0.0412 (4)	
H24	0.5333	0.4084	0.4312	0.049*	
C25	0.47669 (10)	0.23215 (19)	0.41312 (8)	0.0445 (4)	
H25	0.5191	0.1866	0.4013	0.053*	
C26	0.40644 (9)	0.16630 (16)	0.41310 (7)	0.0381 (4)	
H26	0.4011	0.0756	0.4013	0.046*	
O1W	0.5000	−0.1491 (2)	0.2500	0.0825 (8)	
H1W	0.4569	−0.0982	0.2336	0.124*	

### Atomic displacement parameters (Å^2^)

**Table:**

	*U*^11^	*U*^22^	*U*^33^	*U*^12^	*U*^13^	*U*^23^
N1	0.0244 (6)	0.0261 (6)	0.0264 (6)	−0.0000 (5)	0.0040 (5)	−0.0001 (5)
C2	0.0261 (7)	0.0294 (7)	0.0314 (7)	0.0011 (6)	0.0028 (6)	−0.0000 (6)
N3	0.0260 (6)	0.0326 (7)	0.0344 (7)	−0.0007 (5)	0.0059 (5)	−0.0011 (5)
C4	0.0275 (7)	0.0262 (7)	0.0330 (8)	−0.0046 (6)	0.0066 (6)	−0.0010 (6)
C5	0.0321 (8)	0.0326 (8)	0.0389 (8)	−0.0052 (6)	0.0122 (6)	−0.0010 (7)
N6	0.0409 (7)	0.0353 (7)	0.0346 (7)	−0.0064 (6)	0.0126 (6)	0.0017 (6)
C7	0.0394 (8)	0.0260 (7)	0.0304 (8)	−0.0073 (6)	0.0068 (6)	0.0007 (6)
N8	0.0489 (8)	0.0342 (7)	0.0283 (7)	−0.0014 (6)	0.0063 (6)	0.0048 (6)
C9	0.0451 (9)	0.0298 (8)	0.0353 (8)	0.0034 (7)	0.0020 (7)	0.0020 (7)
C10	0.0387 (8)	0.0282 (7)	0.0304 (8)	0.0016 (6)	0.0040 (6)	−0.0004 (6)
C11	0.0330 (7)	0.0233 (7)	0.0288 (7)	−0.0052 (6)	0.0046 (6)	−0.0014 (6)
C12	0.0290 (7)	0.0220 (6)	0.0274 (7)	−0.0062 (5)	0.0056 (5)	−0.0018 (6)
C13	0.0252 (7)	0.0242 (7)	0.0274 (7)	0.0035 (5)	0.0042 (5)	−0.0005 (6)
C14	0.0215 (7)	0.0316 (7)	0.0320 (8)	0.0001 (5)	0.0048 (6)	−0.0044 (6)
C15	0.0279 (7)	0.0256 (7)	0.0424 (9)	−0.0045 (6)	0.0044 (6)	0.0001 (6)
C16	0.0308 (7)	0.0300 (7)	0.0346 (8)	−0.0056 (6)	0.0048 (6)	0.0059 (6)
N17	0.0298 (6)	0.0267 (6)	0.0251 (6)	−0.0058 (5)	0.0027 (5)	0.0018 (5)
C18	0.0308 (7)	0.0233 (7)	0.0281 (7)	−0.0009 (6)	0.0060 (6)	−0.0008 (6)
C19	0.0304 (8)	0.0443 (9)	0.0393 (9)	−0.0065 (7)	0.0069 (6)	−0.0123 (7)
C20	0.0352 (8)	0.0391 (8)	0.0251 (7)	−0.0084 (6)	0.0062 (6)	0.0003 (6)
C21	0.0301 (7)	0.0305 (7)	0.0214 (7)	−0.0027 (6)	0.0013 (5)	0.0016 (6)
C22	0.0343 (8)	0.0326 (8)	0.0262 (7)	−0.0000 (6)	0.0045 (6)	−0.0008 (6)
C23	0.0451 (9)	0.0312 (8)	0.0327 (8)	−0.0095 (7)	0.0003 (7)	0.0007 (7)
C24	0.0325 (8)	0.0513 (10)	0.0379 (9)	−0.0133 (7)	0.0018 (7)	0.0072 (8)
C25	0.0306 (8)	0.0537 (11)	0.0496 (10)	0.0062 (7)	0.0087 (7)	0.0011 (8)
C26	0.0371 (8)	0.0315 (8)	0.0447 (9)	0.0015 (6)	0.0052 (7)	−0.0030 (7)
O1W	0.0391 (11)	0.0429 (11)	0.158 (2)	0.000	0.0001 (12)	0.000

### Geometric parameters (Å, º)

**Table:**

N1—C2	1.3786 (18)	C15—H15A	0.9900
N1—C12	1.3815 (18)	C15—H15B	0.9900
N1—C13	1.4777 (17)	C16—N17	1.4667 (17)
C2—N3	1.3132 (18)	C16—H16A	0.9900
C2—H2	0.9500	C16—H16B	0.9900
N3—C4	1.3936 (19)	N17—C18	1.4642 (18)
C4—C12	1.401 (2)	N17—C20	1.4654 (18)
C4—C5	1.407 (2)	C18—H18A	0.9900
C5—N6	1.332 (2)	C18—H18B	0.9900
C5—H5	0.9500	C19—H19A	0.9800
N6—C7	1.348 (2)	C19—H19B	0.9800
C7—N8	1.378 (2)	C19—H19C	0.9800
C7—C11	1.419 (2)	C20—C21	1.512 (2)
N8—C9	1.368 (2)	C20—H20A	0.9900
N8—H8	0.9793	C20—H20B	0.9900
C9—C10	1.368 (2)	C21—C22	1.385 (2)
C9—H9	0.9500	C21—C26	1.391 (2)
C10—C11	1.432 (2)	C22—C23	1.386 (2)
C10—H10	0.9500	C22—H22	0.9500
C11—C12	1.408 (2)	C23—C24	1.376 (2)
C13—C18	1.5234 (19)	C23—H23	0.9500
C13—C14	1.5414 (19)	C24—C25	1.379 (3)
C13—H13	1.0000	C24—H24	0.9500
C14—C15	1.526 (2)	C25—C26	1.387 (2)
C14—C19	1.528 (2)	C25—H25	0.9500
C14—H14	1.0000	C26—H26	0.9500
C15—C16	1.518 (2)	O1W—H1W	0.9253
			
C2—N1—C12	105.96 (11)	C14—C15—H15B	109.2
C2—N1—C13	128.98 (12)	H15A—C15—H15B	107.9
C12—N1—C13	125.05 (11)	N17—C16—C15	109.69 (12)
N3—C2—N1	113.88 (13)	N17—C16—H16A	109.7
N3—C2—H2	123.1	C15—C16—H16A	109.7
N1—C2—H2	123.1	N17—C16—H16B	109.7
C2—N3—C4	104.26 (12)	C15—C16—H16B	109.7
N3—C4—C12	110.30 (12)	H16A—C16—H16B	108.2
N3—C4—C5	129.35 (13)	C18—N17—C20	110.45 (11)
C12—C4—C5	120.31 (13)	C18—N17—C16	109.44 (11)
N6—C5—C4	122.23 (14)	C20—N17—C16	110.67 (11)
N6—C5—H5	118.9	N17—C18—C13	112.78 (11)
C4—C5—H5	118.9	N17—C18—H18A	109.0
C5—N6—C7	115.68 (13)	C13—C18—H18A	109.0
N6—C7—N8	123.80 (13)	N17—C18—H18B	109.0
N6—C7—C11	128.67 (14)	C13—C18—H18B	109.0
N8—C7—C11	107.52 (13)	H18A—C18—H18B	107.8
C9—N8—C7	108.42 (13)	C14—C19—H19A	109.5
C9—N8—H8	126.0	C14—C19—H19B	109.5
C7—N8—H8	125.6	H19A—C19—H19B	109.5
N8—C9—C10	110.92 (14)	C14—C19—H19C	109.5
N8—C9—H9	124.5	H19A—C19—H19C	109.5
C10—C9—H9	124.5	H19B—C19—H19C	109.5
C9—C10—C11	106.11 (14)	N17—C20—C21	112.72 (11)
C9—C10—H10	126.9	N17—C20—H20A	109.0
C11—C10—H10	126.9	C21—C20—H20A	109.0
C12—C11—C7	113.22 (13)	N17—C20—H20B	109.0
C12—C11—C10	139.75 (14)	C21—C20—H20B	109.0
C7—C11—C10	107.03 (13)	H20A—C20—H20B	107.8
N1—C12—C4	105.59 (12)	C22—C21—C26	118.41 (14)
N1—C12—C11	134.53 (13)	C22—C21—C20	121.30 (13)
C4—C12—C11	119.86 (13)	C26—C21—C20	120.28 (13)
N1—C13—C18	111.55 (11)	C21—C22—C23	120.92 (14)
N1—C13—C14	112.57 (11)	C21—C22—H22	119.5
C18—C13—C14	111.61 (11)	C23—C22—H22	119.5
N1—C13—H13	106.9	C24—C23—C22	120.00 (15)
C18—C13—H13	106.9	C24—C23—H23	120.0
C14—C13—H13	106.9	C22—C23—H23	120.0
C15—C14—C19	111.95 (12)	C23—C24—C25	119.98 (15)
C15—C14—C13	111.12 (11)	C23—C24—H24	120.0
C19—C14—C13	112.55 (12)	C25—C24—H24	120.0
C15—C14—H14	106.9	C24—C25—C26	119.96 (16)
C19—C14—H14	106.9	C24—C25—H25	120.0
C13—C14—H14	106.9	C26—C25—H25	120.0
C16—C15—C14	112.05 (12)	C25—C26—C21	120.70 (15)
C16—C15—H15A	109.2	C25—C26—H26	119.6
C14—C15—H15A	109.2	C21—C26—H26	119.6
C16—C15—H15B	109.2		
			
C12—N1—C2—N3	0.50 (16)	C10—C11—C12—C4	178.39 (16)
C13—N1—C2—N3	179.23 (12)	C2—N1—C13—C18	46.14 (18)
N1—C2—N3—C4	−0.21 (16)	C12—N1—C13—C18	−135.35 (13)
C2—N3—C4—C12	−0.15 (15)	C2—N1—C13—C14	−80.23 (17)
C2—N3—C4—C5	177.61 (15)	C12—N1—C13—C14	98.28 (15)
N3—C4—C5—N6	−176.99 (14)	N1—C13—C14—C15	80.55 (14)
C12—C4—C5—N6	0.6 (2)	C18—C13—C14—C15	−45.79 (15)
C4—C5—N6—C7	−1.4 (2)	N1—C13—C14—C19	−45.91 (16)
C5—N6—C7—N8	−178.11 (14)	C18—C13—C14—C19	−172.24 (12)
C5—N6—C7—C11	0.6 (2)	C19—C14—C15—C16	176.63 (12)
N6—C7—N8—C9	179.33 (14)	C13—C14—C15—C16	49.84 (15)
C11—C7—N8—C9	0.35 (16)	C14—C15—C16—N17	−58.88 (15)
C7—N8—C9—C10	−0.34 (18)	C15—C16—N17—C18	63.33 (15)
N8—C9—C10—C11	0.18 (18)	C15—C16—N17—C20	−174.72 (12)
N6—C7—C11—C12	0.9 (2)	C20—N17—C18—C13	176.90 (11)
N8—C7—C11—C12	179.84 (12)	C16—N17—C18—C13	−61.02 (14)
N6—C7—C11—C10	−179.16 (14)	N1—C13—C18—N17	−74.69 (14)
N8—C7—C11—C10	−0.24 (16)	C14—C13—C18—N17	52.20 (15)
C9—C10—C11—C12	179.93 (17)	C18—N17—C20—C21	−70.56 (15)
C9—C10—C11—C7	0.04 (16)	C16—N17—C20—C21	168.08 (12)
C2—N1—C12—C4	−0.54 (14)	N17—C20—C21—C22	124.87 (14)
C13—N1—C12—C4	−179.34 (12)	N17—C20—C21—C26	−55.85 (18)
C2—N1—C12—C11	−178.93 (15)	C26—C21—C22—C23	−1.7 (2)
C13—N1—C12—C11	2.3 (2)	C20—C21—C22—C23	177.56 (13)
N3—C4—C12—N1	0.44 (15)	C21—C22—C23—C24	0.7 (2)
C5—C4—C12—N1	−177.55 (12)	C22—C23—C24—C25	0.9 (2)
N3—C4—C12—C11	179.12 (12)	C23—C24—C25—C26	−1.3 (3)
C5—C4—C12—C11	1.1 (2)	C24—C25—C26—C21	0.2 (3)
C7—C11—C12—N1	176.48 (14)	C22—C21—C26—C25	1.3 (2)
C10—C11—C12—N1	−3.4 (3)	C20—C21—C26—C25	−178.01 (14)
C7—C11—C12—C4	−1.73 (18)		

### Hydrogen-bond geometry (Å, º)

**Table:**

*D*—H···*A*	*D*—H	H···*A*	*D*···*A*	*D*—H···*A*
N8—H8···N6^i^	0.98	2.02	2.9757 (18)	165
O1*W*—H1*W*···N3	0.93	2.13	3.0148 (16)	161

Symmetry code: (i) −*x*+1/2, −*y*+1/2, −*z*.
